# Screening for Toxic Amyloid in Yeast Exemplifies the Role of Alternative Pathway Responsible for Cytotoxicity

**DOI:** 10.1371/journal.pone.0004539

**Published:** 2009-03-05

**Authors:** Julien Couthouis, Karine Rébora, Françoise Immel, Karine Berthelot, Michel Castroviejo, Christophe Cullin

**Affiliations:** 1 IBGC, UMR 5095, CNRS Université Bordeaux 2 “Victor Segalen”, Bordeaux, France; 2 REGER, UMR 5097 CNRS Université Bordeaux 2 “Victor Segalen”, Bordeaux, France; Columbia University, United States of America

## Abstract

The relationship between amyloid and toxic species is a central problem since the discovery of amyloid structures in different diseases. Despite intensive efforts in the field, the deleterious species remains unknown at the molecular level. This may reflect the lack of any structure-toxicity study based on a genetic approach. Here we show that a structure-toxicity study without any biochemical prerequisite can be successfully achieved in yeast. A PCR mutagenesis of the amyloid domain of HET-s leads to the identification of a mutant that might impair cellular viability. Cellular and biochemical analyses demonstrate that this toxic mutant forms GFP-amyloid aggregates that differ from the wild-type aggregates in their shape, size and molecular organization. The chaperone Hsp104 that helps to disassemble protein aggregates is strictly required for the cellular toxicity. Our structure-toxicity study suggests that the smallest aggregates are the most toxic, and opens a new way to analyze the relationship between structure and toxicity of amyloid species.

## Introduction

The link between aggregated proteins and toxic species stems from earlier studies on neurodegenerative diseases. This relationship was initially assessed by the presence of proteinaceous deposits in the brain of patients who suffered from such disease. The particular aggregation found in amyloid structures results from protein assembly into fibrils that exhibit cross-ß diffraction pattern, ß-sheet-rich CD (Circular Dichroism) and FTIR (Fourier Transform Infra-Red) spectra, core structure highly resistant to proteases and metachromic properties. Our knowledge of these particular aggregates is the outcome of different disciplines including genetics, physiology, biochemistry, cell biology and biophysics (for a review, see [1], [2]). Initial interest for amyloid structures comes obviously from their link with the complex phenomena that leads to neurodegeneration and disease. Two fields of interest, *in vitro* or *in vivo*, have evolved in parallel, but the interconnection of the data in a unified scheme is tricky, so the determination of the initial events and the main actors involved in this cascade is difficult. Moreover, most if not all the *in vitro* approaches are centered on the polymerization mechanism and cannot directly help to understand the way by which cellular toxicity is achieved. The existence of mutated amyloid proteins that cause susceptibility to disease has permitted to link the polymerization characteristics with the pathogenesis [3], [4]. Despite intensive research in the field, no one has ever screened a randomly generated library of amyloid protein for its toxic capacity, making hazardous the establishment of general rules that would connect amyloid polymerization and cellular toxicity. One of the difficulties comes from the capacity to screen such library.

The budding yeast offers a convenient system to monitor amyloid toxicity [5]–[7] and has allowed in the past pinpointing genes that modulate the deleterious consequences in other eukaryotic models [8]. Since the yeast model offers a convenient system to identify genes involved in *trans* in amyloid toxicity, we decided to use it as the host for the identification of the *cis* mutations that make toxic a benign amyloid. The amyloid model used in this study is the prion domain of a fungal prion protein: HET-s [9]. This 72 amino acids peptide forms the proteinase K-resistant core of the prion fibrils made with the HET-s protein. This C-terminal domain is unstructured in solution and forms infectious amyloid fibrils *in vitro* [10]. This protein has been chosen since a molecular model for amyloid assembly [11] has been proposed on the ground of NMR data. This organization implies 4 ß-sheets in parallel constituted by two pseudo-repeat sequences, each forming a ß-strand-turn-ß-strand motif. More recently, a solid-state NMR study has revealed that HET-s (218–289) forms a left-handed ß solenoid in which each previous ß-strand is split into two shorter segments [12]. This protein has been successfully expressed in yeast cells as a chimeric HET-s-GFP protein. Moreover, this protein produces infectious amyloid-like aggregates in *Saccharomyces cerevisiae* [13] and thus, offers a convenient “starting-point” for our study since this amyloid does not impair the yeast cellular viability.

In this article, we have selected toxic species after an error-prone PCR mutagenesis of HET-s (218–289). The most toxic mutant (m8) clearly exhibits a different pattern of aggregation. We found that this mutant forms smaller aggregates that appear to belong to a different aggregation pathway controlled by Hsp104, a key factor in aggregative mechanisms in yeast.

## Results

### Screening for toxic species revealed different cellular patterns of aggregation

**Figure 1:**
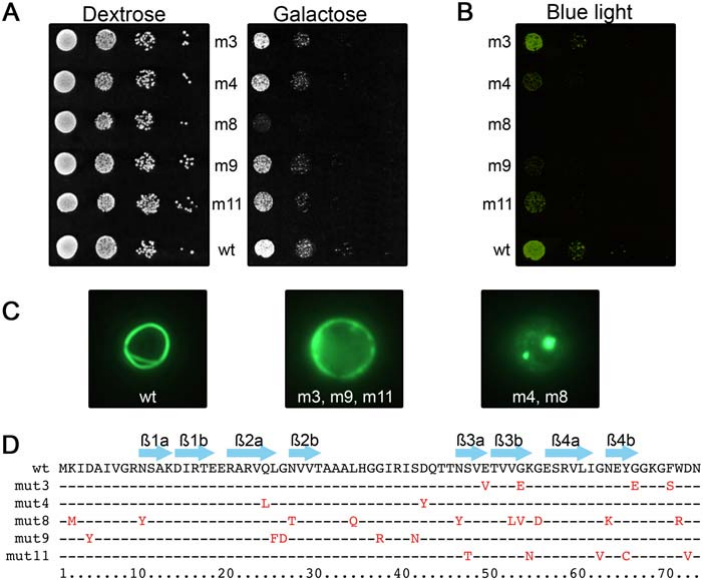
Identification of PCR-engineered toxic alleles of HET-s(PrD). (A) Mutants exhibit a wide range of toxicity in yeast. A ten-fold serial dilution of the various transformants was analyzed in dextrose (repression condition) or galactose (induction condition). The growth of cells was observed after 48 h. (B) Mutants exhibit a wide range of expression levels in yeast. The galactose plate of panel (A) was observed using a Safe Imager™ blue light transilluminator and Safe Imager™ amber filter (Invitrogen) to visualize differences in GFP-fluorescence between the various PCR-engineered HET-s(PrD) mutants. (C) Mutants exhibit different GFP aggregation patterns in yeast. Cells were examined under a fluorescence microscope after 72 h of growth in liquid galactose medium. (D) Amino acid sequence alignment of wild-type and mutant HET-s(PrD) domains. Arrows above the sequence outline the ß-strands.

HET-s, a protein of *Podospora anserina* is able to form *in vivo* a prion [9] that switches into its infectious form after being exposed to *in vitro*-formed fibers of purified PrD fragments of HET-s [14]. In this study, we used a previously engineered plasmid (pYecHetsYGFP2U) that contains the prion domain (PrD) of HET-s fused to the coding sequence of the GFP under the control of a galactose-inducible promoter [13]. Several mutants of HET-s(218–289) have been generated by a PCR mutagenesis and introduced in this plasmid to replace the wild-type sequence of HET-s(PrD). A first screen led us to identify 80 clones among more than 20 000 transformants that grew more slowly than the control containing the wild-type HET-s(218–289)-GFP. The putative toxicity of these HET-s(PrD)-GFP mutants was confirmed by testing their growth in a spotting assay. After two days on a galactose medium (induction conditions), differences in the growth of strains containing wild-type and mutated PrD were clearly confirmed for five mutants whereas they grew similarly on the glucose medium (repression conditions). Only one of them, m8, led to a more severe growth defect, while the others displayed gradual levels of toxicity (Figure 1A). The amount of fusion-GFP protein produced in these strains can be directly appreciated by visualizing the fluorescence intensity after exposure to a 470 nm transilluminator. We thus verified that the level of toxicity was not correlated to a higher amount of GFP species formed (Figure 1B). When cells in stationary phase were observed under a fluorescence microscope, the aggregates formed by the mutants appear to be different from those formed by the wt protein. The aggregation patterns fall into three groups (Figure 1C): wt forms typical rings, m8 and m4 form bright dots and m3, m9 and m11 present a diffuse fluorescence. These differences may reflect either structural modifications or changes of the interaction between the HET-s(PrD)-GFP protein and cellular partners. The mutated residues were found all along the sequence (Figure 1D) and not only located in the domains previously proposed to form ß-sheets in the fibrils [11], [12]. By mutating one or several residues of HET-s(218–289), we were thus able to generate some toxic forms, that exhibit a different GFP aggregation pattern, as shown by microscopy.

### Identification of mutations essential for toxicity

**Figure 2:**
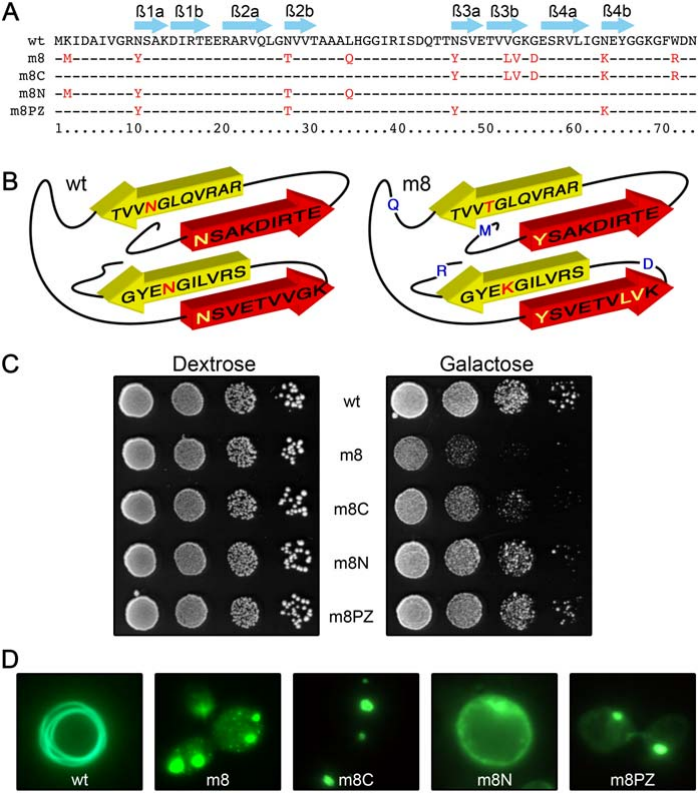
Identification of mutations required to induce toxicity. (A) Amino acid sequence alignment of wild-type, m8 and chimeric mutants of HET-s(PrD) domains. Arrows above the sequence outline the ß-strands. (B) 3D modeling of HET-s(PrD) domain. Mutated amino acid in m8 (right panel) and wild-type (left panel) are located on a basic 3D model according to the RMN structure prediction [12]. (C) A ten-fold serial dilution of the various transformants was placed on solid dextrose or galactose medium and the growth of cells was observed after 48 h. (D) Mutants exhibit different GFP aggregation patterns in yeast. Cells were examined under a fluorescence microscope after 72 h of growth in liquid galactose medium.

Despite intensive screening, we only got a few mutants (an independent screening of additional 20 000 transformants did not allow to isolate new toxic mutants, data not shown). Only one of the five mutants isolated (the m8 mutant) shows a toxic phenotype strong enough to allow a further characterization. This mutant possesses ten mutations located all along the primary sequence (Figure 2A). In order to identify which of these mutation(s) was responsible for the toxic phenotype, we have engineered new alleles of HET-s(PrD)-GFP that bear only some of the mutations found in m8. The m8N chimeric protein includes the first four mutations spanning from residues 1 to 38, whereas the reciprocal m8C protein contains the six mutations located at the C terminus (Figure 2A). These two mutants are interesting since they bear the mutations in each of the two layers of ß strand-turn-ß strand motifs of HET-s (218–289) [11], [12] (Figure 2B). In a spotting assay, m8N allele behaves as the wt in yeast cells. The m8C is slightly toxic but not as much the m8 (Figure 2C). These results indicate that, in m8, more than one mutation is required to switch from benign to highly toxic species. Moreover, these mutations have to be located in the two elementary motifs that are interconnected by a loop. This may explain the relative scarcity of the toxic mutants isolated from the screen. We have then analyzed the mutations located in the ß strands. Interestingly, four of these six mutations concern four asparagines that are mutated in m8. The position of these asparagines in the structural model [12] is compatible with the formation of a polar zipper already described for glutamines and asparagines [15], [16] (Figure S1). As such polar zipper may be crucial for the stabilization of the amyloid fibril, we have generated a new allele (m8PZ) in which these four asparagines were selectively replaced by the four amino acids found in m8 (Figure 2A). When expressed in yeast cells, this new mutant behaves as the wild-type since it does not impair the cellular viability (Figure 2C).

Interestingly, these three additional mutants do not aggregate in yeast cells as the wt since they form fluorescent foci instead of the characteristic annular morphology (Figure 2D). This punctuate phenotype observed with a fluorescence microscope is therefore not sufficient by itself to predict the toxicity, but rather represents a first level of characterization of the aggregates.

### The most toxic mutant disrupts the cell-cycle

**Figure 3:**
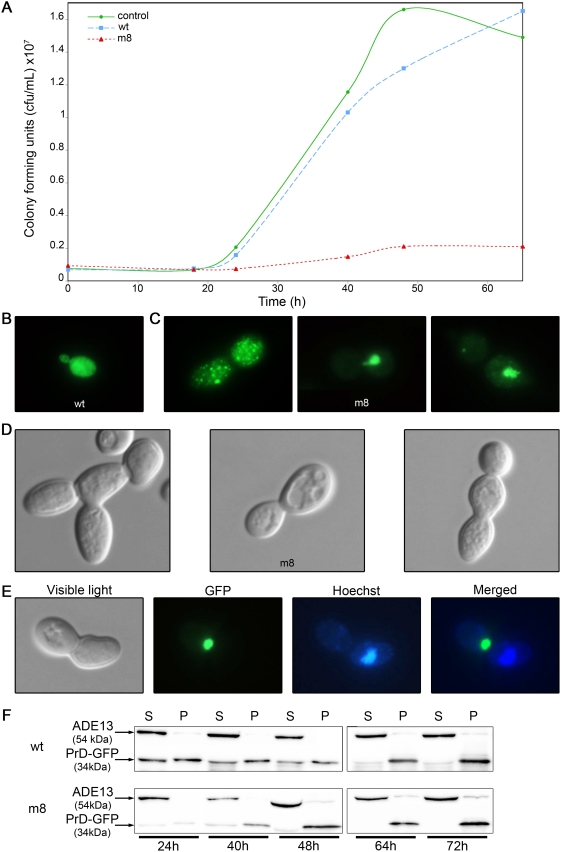
The m8 HET-s(PrD)-GFP mutant impairs cell viability. (A) Toxicity of m8 expression demonstrated by a viability assay. The strain BY4742 was transformed with a plasmid expressing either wt or m8 HET-s(PrD)-GFP or a control plasmid. Liquid galactose medium was then inoculated and grown for 18 to 65 hours before being plated onto glucose solid medium. Colonies were numbered, and this value was then converted to colony forming units (cfu/mL). (B) Cells expressing wild-type HET-s(PrD)-GFP were examined with a fluorescence microscope after 24 h of growth in liquid galactose medium. (C) Cells expressing m8 HET-s(PrD)-GFP were observed using a fluorescence microscope after 24 h of growth in liquid galactose medium. (D) Cells expressing m8 HET-s(PrD)-GFP were observed with a light microscope after 24 h of growth in liquid galactose medium, using a Nomarski contrast filter. (E) Cells expressing m8 HET-s(PrD)-GFP were examined using both a light and a fluorescence microscope after 24 h of growth in liquid galactose medium. GFP fluorescence (green) and Hoechst DNA staining (blue) of an isolated dividing cell are shown. A GFP/DNA merged image was made using Adobe Photoshop. (F) Distribution of the GFP species between pellet (P) and supernatant (S). An aliquot of the culture used in the previous cfu assay was pipetted out after 24 to 72 hours. Total cell extracts underwent ultracentrifugation for 30 min; supernatant and pellet were run on a 12% SDS-PAGE gel, and were transferred onto a nitrocellulose membrane and exposed to anti-GFP antibodies.

We then focused on m8, the most toxic mutant selected. To investigate the toxicity of the m8 protein, we estimated the number of colony forming units (cfu) in a liquid culture at different times after expression of the protein (Figure 3A). The number of cfu in the culture of cells expressing the m8 protein hardly increased during the experiment, whereas after a lag phase of 24 h, it increased for cells expressing the wt HET-s(PrD). Since the differences occur when the cells restart their cellular division, m8 protein could affects the cell viability as well as the division capacity. A Trypan blue staining of cells reveals that the percentage of stained cells is similar in cells expressing the wt or m8 protein after 28 h of expression, whereas at 54 h, there are three times more stained cells among the cells expressing m8 that among the cells expressing wt (data not shown). This suggests then that after 24 h of induction, the expression of the m8 protein has affected the division ability of the cells, but not yet their viability. At that time, cells expressing m8 show a GFP profile very different from the wt in exponential phase. The wt protein is evenly distributed throughout the cytoplasm of the yeast cells (Figure 3B), whereas the m8 protein forms one large or numerous small dots (Figure 3C). Furthermore, some cells expressing the m8 mutant show an abnormal morphology (Figure 3D): a large number of cells are distorted, and strings of connected budding cells (often three) are observed, indicating a possible link with a defect in the cytokinesis and/or cell polarity. A commonly observed pattern is shown in the figure 3E: two cells are attached, one of them presenting a large dot. Hoechst staining of the DNA showed that only one of the two cells has a nucleus, the one that does not have the aggregate (merged image), suggesting that aggregation of the m8 protein has caused a defect in nuclear division in such cells. A Western-blot was made on the same culture with an anti-GFP antibody (Figure 3F). The wt protein is expressed at its maximum level at 24 h and equally distributed between the supernatant and the 100,000 g pellet. The pellet/supernatant ratio increases during the induction up to 64 h. The m8 protein is only weakly expressed at 24 h, and reaches its maximum level of expression after 48 h of induction (Figure 3F). Most of the protein is located in the pellet, as expected since the presence of foci is observed in fluorescence microscopy. Our results thus clearly show that m8-PrD-GFP affects cell fitness and exhibits a different cellular pattern of aggregation.

### The toxic aggregates differ from the wild-type and non toxic punctuate aggregates

**Figure 4:**
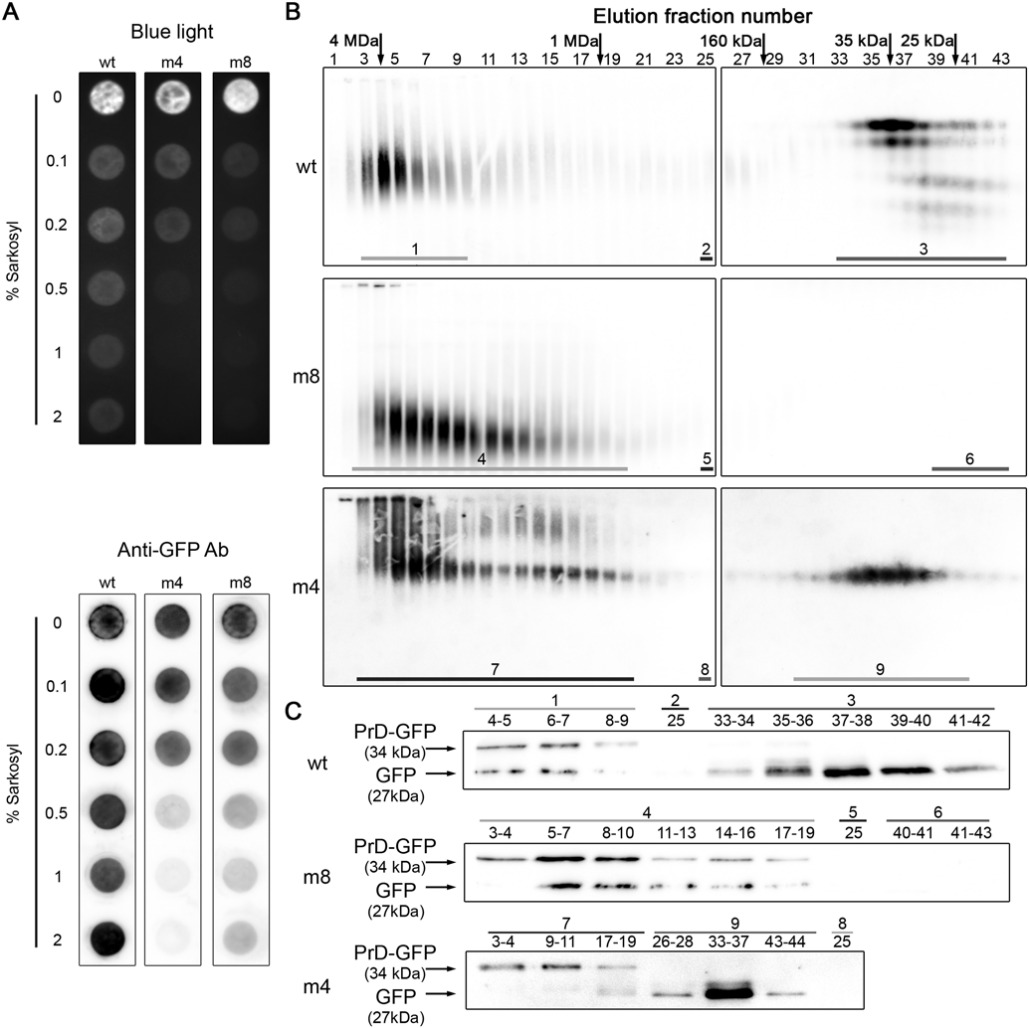
Size distribution and detergent resistance of Wild-type, m4 and m8 HET-s(PrD)-GFP aggregates. Total extracts from cells expressing wt, m4 or m8 HET-s(PrD)-GFP were prepared after 24 h of growth in liquid galactose medium. (A) Filter trap assay of wt, m4 and m8 crude extracts through a cellulose acetate membrane after incubation in various sarkosyl concentrations (0 to 2%) as indicated. The membrane was visualized with a Safe Imager™ blue light transilluminator and Safe Imager™ amber filter (Invitrogen) (upper panel), it was then blotted with anti-GFP antibodies and analyzed using the VersaDoc Imaging System (BioRad) (lower panel). (B) Analysis of all fractions resulting from the SEC experiment on 2.5% native agarose gel visualized by western blot with anti-GFP antibodies. (C) Analysis of several fractions underlined in (B) on a 12% SDS PAGE followed by western-blot.

To biochemically characterize these aggregates; a filter trap assay was first made on crude extracts. The GFP-aggregates retained on the filter were detected using a blue light transilluminator (Figure 4A upper) and blotted with an anti-GFP antibody (Figure 4A lower). As the m4 mutant (which is slightly toxic) exhibits the same pattern of cytosolic aggregates when observed with a fluorescence microscope, it was also of interest to compare its behavior in the same assay. The presence of a signal is observed for extracts from cells expressing either wt, m4 or m8 proteins, indicating the presence of large protein aggregates (over 0.2 µm) (Figure 4A). The stability of such aggregates can be monitored by their sensitivity to detergents. The m8 aggregates appeared to be more sensitive than the wt to sarkosyl (an anionic mild detergent) since the signal decreases more strongly as the concentration of detergent increased (Figure 4A). In the same conditions, m4 aggregates behave as m8 aggregates. They are solubilized (ie are no more retained onto the surface of a 0.2 µm membrane) more easily than the wt when the sarkosyl reaches 0.5%. To analyze the size distribution of smaller aggregates, crude extracts of cells expressing either wt, m4 or m8 proteins were then fractionated by size-exclusion chromatography after filtration on a 0.2 µm membrane in the presence of 0.1% sarkosyl. The different fractions were then resolved on a native agarose gel and probed with anti-GFP antibodies (Figure 4B). In each case, we observed a first “wave” of GFP species corresponding to large aggregates (fraction 4 and 18 correspond to 4 MDa and 1 MDa size marker respectively). This first wave is wider for the m4 and m8 protein, which means that these aggregates go over a larger range of size (from 4 MDa to 1 MDa), going down to smaller aggregates. For the wt, the smear is centered on the same value in all the fractions (from 1 to 18) in which the GFP can be detected. This is consistent with a Gaussian distribution of the same entities. The differences observed between m8 and wt suggest that m8 may form smaller aggregates than the wt protein. *In vitro*, m8 assemble into typical small amyloid fibrils that do not exceed 80 nm of length [17]. This capacity to form smaller aggregates is therefore consistent with the presence of smaller aggregates *in vivo*.

Western blot analysis of several fractions in presence of SDS (Figure 4C) indicates the presence of two bands, one corresponding to HET-s(PrD)-GFP protein, and a smaller species that is likely to be the GFP moiety, according to its size. The native gel electrophoresis probed with anti-GFP antibodies also reveals the presence of GFP entities in the last fractions of wt and m4 elutions (from fraction 33 to 43). This signal is not detected for m8. Western blot analysis in denaturing conditions of these fractions clearly indicates the presence of only one band that corresponds to the GFP moiety, according to its size. In wt extract, this protein migrates very high in the native gel (Figure 4C), much higher than the protein present in the high molecular weight fraction, whereas in m4 extracts, this protein migrates more rapidly. In these conditions, there is clearly no correlation between the distance of migration and the size of the species, probably because of the differences in the charge of the protein species between soluble and aggregated forms. The presence of the GFP moiety may be due either to a cleavage in the crude extract during the SEC experiment or may reflect a proteolysis that occurs *in vivo*. A rapid alkaline lysis of the cells followed by a TCA precipitation, SDS PAGE and western-blot reveals that m8 and HET-s(PrD)-GFP exhibit a small amount of intracellular cleavage (Figure S2). This result indicates that the presence of the GFP without its amyloid domain is mainly due to a proteolysis that occurs during SEC experiment.

These results show that wt and m8 proteins form two aggregates that differ in their size (the wt protein forming on average larger aggregates than m8) and in their biochemical properties (resistance to detergent). The non-toxic m4 mutant shares the same sensitivity to sarkosyl than the m8, but the aggregates separated by the SEC are clearly different.

### Hsp104 acts as trans factor on m8 toxicity

**Figure 5:**
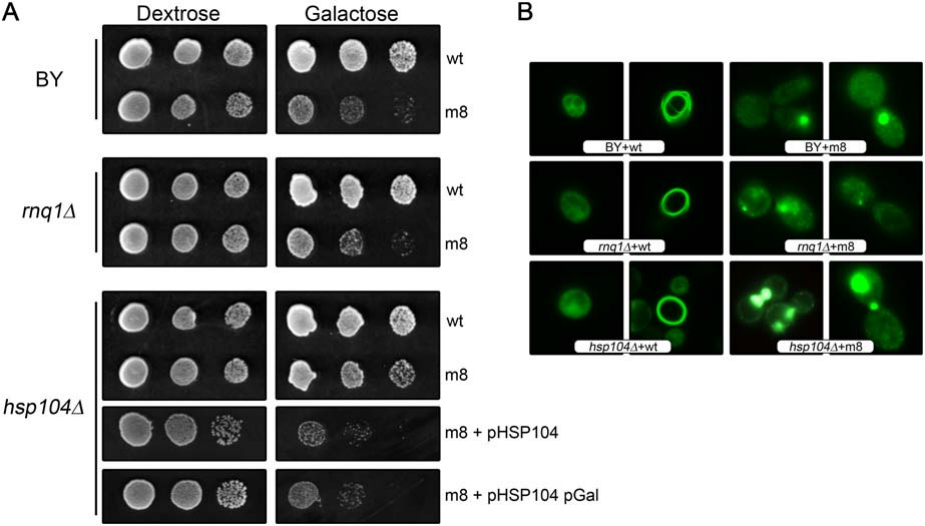
*Trans* modulating effects on m8 HET-s(PrD)-GFP toxicity. (A) Wild-type, *rnq1Δ* and *hsp104Δ* strains were transformed with a plasmid expressing either wt or m8 HET-s(PrD)-GFP. The *hsp104Δ* was also co-transformed with a 2 µ plasmid bearing either wt *HSP104* gene (pHSP104 plasmid) or with a galactose inducible *HSP104* allele (pHSP104 pGal plasmid). The toxicity was tested as in Fig. 1. (B) Cells were examined under a fluorescence microscope after 48 h of growth in liquid galactose medium.

As Rnq1p and Hsp104 have been previously shown to be critical for the toxicity and the aggregation of the poly-glutamine amyloid protein in yeast [5], we tested if they were necessary for the toxicity and the aggregation of the m8 fusion protein. The expression of the m8 protein was still toxic in an *rnq1Δ* strain (Figure 5A), and the GFP pattern in this strain was similar to the one observed in a wild-type strain (Figure 5B) which shows that Rnq1p is not involved in the aggregation and the toxicity of m8. On the contrary, the expression of the m8 protein in an *hsp104Δ* strain is not lethal anymore (Figure 5A). The GFP pattern in this strain is quite similar that in a wild-type strain, with a tendency to make more big dots and less small grains (Figure 5B). This shows that Hsp104 plays a role in the toxicity of the m8 protein, maybe by favoring the conversion of big aggregates into smaller and toxic ones. It is also an indirect proof showing that the toxic species is indeed related to the aggregation process. However, the role of Hsp104 could be indirect as it is the case for polyQ aggregates that required the prion [RNQ1+] to be toxic. To test this hypothesis, we have introduced a multi copy plasmid containing the wild-type *HSP104* gene in an *hsp104Δ* strain already transformed by pYem8YGFP2U (that allows m8 expression). In these conditions, the toxicity was restored (Figure 5A). As this toxicity could be due to a prion that would appear during the 15–16 generations required to get the transformant, we used an additional strategy. A plasmid bearing a chimeric gene in which the coding sequence of *HSP104* is under the control of the *GAL10* promoter was used to transform an *hsp104Δ* strain already transformed by pYem8YGFP2U. When this strain was spotted into a medium allowing the expression of m8 and Hsp104, the growth was clearly impaired (Figure 5A). Since Hsp104 is produced in these conditions in a couple of hours, the re-appearance of a prion during this time is very unlikely and our results are consistent with a role of Hsp104 as a direct modulator of the m8 toxicity.

### Relationship between m8 and wt aggregates

**Figure 6:**
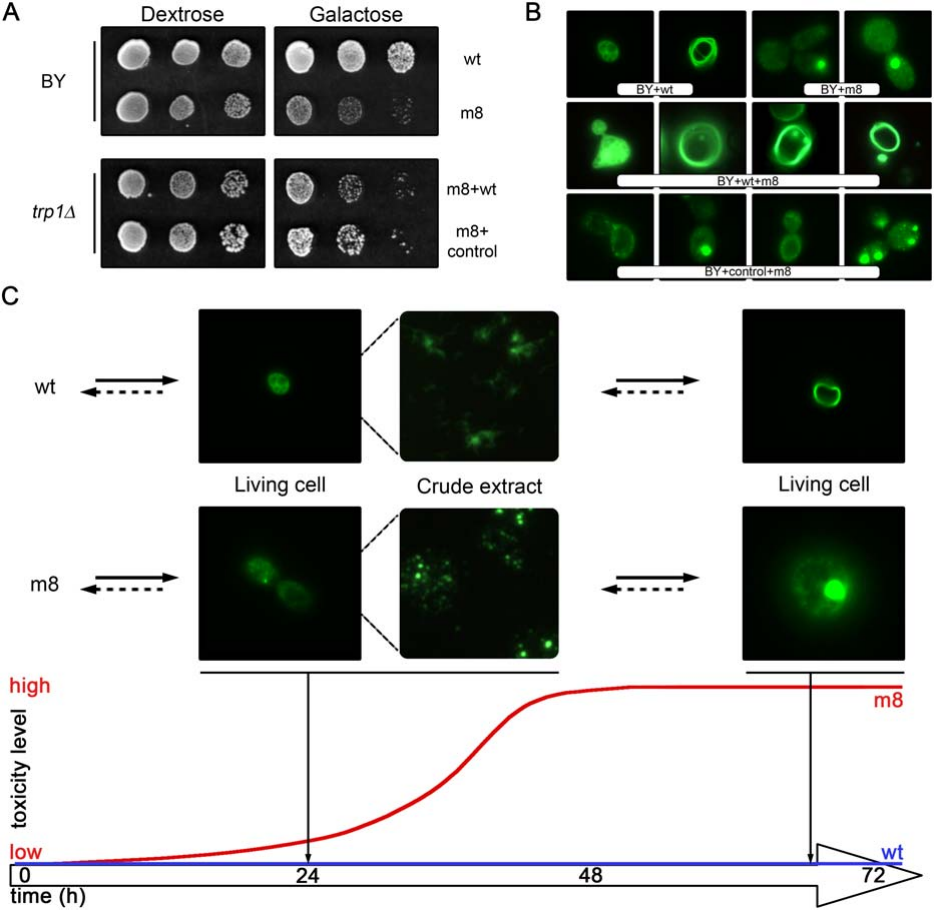
Aggregation pathways of Wild-type and m8 HET-s(PrD)-GFP. (A) Expression of wt HET-s(PrD)-GFP does not rescue m8 toxicity. A ten-fold serial dilution of the strains co-transformed with m8 HET-s(PrD)-GFP and either wt HET-s(PrD)-GFP or control plasmid was analyzed as in Fig. 1. (B) Cells were examined under a fluorescence microscope after 48 h of growth in liquid galactose medium. (C) Modeling of the two potential aggregation pathways. The crude extracts used in the size-exclusion chromatography were visualized with the fluorescence microscope. Wild-type HET-s(PrD)-GFP forms cottony aggregates, whereas m8 HET-s(PrD)-GFP forms dot-shaped aggregates. These GFP aggregation patterns are scaled on a time and toxicity curve to summarize our experimental results.

Two hypotheses can explain the difference of toxicity between m8 and wt proteins. In the first case, the toxic species that make m8 harmful would exist (but in lower concentration) in yeast cells expressing the wt amyloid (because they would be in equilibrium with other non-toxic species) or alternatively these toxic species would arise by a distinct pathway and are specific to m8. To answer this question, we coexpressed both proteins in a wild-type yeast strain. We assumed that if the toxic species are part of the same pathway, the wt protein might titrate the toxic species formed by the m8 protein. In this case, the coexpression would lead to a non-toxic phenotype. However, the expression of the wt protein does not suppress the toxicity of the m8 protein (Figure 6A) and when observed with a fluorescence microscope, we observed some cells containing both m8 characteristic dots and wt characteristic rings (Figure 6B). These results thus suggest that m8 and wt proteins have two independent aggregation pathways leading to different “terminal” products (ring or dots) that may co-exist.

We then proposed a model to summarize our results and explain the differences in the toxicity and the aggregation of the two proteins (Figure 6C). The wt protein, which is not toxic, has a diffuse GFP pattern in cells observed in exponential growing phase, but appears aggregated in a sedimentation assay and when a crude extract from similar cells is observed in fluorescence microscopy, the protein actually makes small fibrils that give a “cottony” appearance (Figure 6C). In stationary phase, it forms typical ring structures. The m8 protein has a very different pattern: during exponential growing phase (*in vivo*) cells or in a crude extract (*in vitro*), it forms small grains or small dots, different from the fibrils observed in the wt. In stationary phase cells, the protein makes bigger dots. Its toxic effect is observed after 36 to 48 h in cells. At this stage, one can detect the presence of smaller aggregated species with the m8, as suggested by our biochemical analyses. In this model (the toxic species being an intermediate in the aggregation process), some factors such as Hsp104 could help or prevent the toxicity by favoring one or several steps of the aggregation process.

## Discussion

Amyloid proteins are not only associated with disease but are also related to various cellular processes. Cells use amyloid proteins for cellular function. *E.coli*, for example, is able to form amyloid fibrils, termed curli [18] which are the major proteinaceous component of a complex extra-cellular matrix and are involved in the colonization of a diverse range of surfaces, such as plant tissues, glass, etc… [19] Another prokaryote (*Streptomyces coelicolor*, a filamentous bacteria) forms amyloid structures that are required for aerial hyphae formation [20]. In eukaryotic cells, amyloids are not systematically associated with disease. In *S. cerevisiae*, at least three proteins (Ure2p, Sup35p and Rnq1p) are able to form *in vitro* amyloid structures, permitting a phenotypic switch if introduced into yeast cells [21]–[24]. R. Wickner coined the word “prion” in 1994 [25] to describe [URE3], the loss of function phenotype due to the aggregation of Ure2p. Yeast and mammalian prions differ fundamentally by their effect on organism viability. The mammalian prion protein Prp is the only infectious amyloid protein found in metazoans (although amyloid A is also suspected to be an infectious amyloid [26], [27]). The other proteins that form *in vivo* amyloid structures in mammals are generally related to a pathological event. In yeast, prions were initially described as a phenotypic switch that does not lead to the death of this organism. Even if the presence of such proteins may be deleterious in many natural [28] or over-expressing [29] conditions some recent studies show that yeast prions may be involved in a fast adaptation mechanism in stress conditions [30]. Human diseases associated with the formation of extracellular amyloid deposits or intracellular inclusions with amyloid-like characteristics have been discussed in a recent review [1]. Interestingly, several of these proteins (both mammalian and fungal) may assemble into oligomeric species that share a common epitope [31]–[33], and these species may be responsible for cellular toxicity [34], [35]. These findings are *paradoxical*, as both benign and toxic amyloid may form toxic species. Why is it that some amyloids are harmful whereas others are harmless? Thus, the question arises of whether it is due to a difference in the rate of toxic intermediate formation or due to the existence of alternative pathways. In other words, does a change between benign and toxic amyloid imply a change in the kinetics of aggregate formation or imply a change in the kind of aggregates formed? Several mutations that are responsible for early onset forms of Alzheimer or Parkinson disease have been isolated in the coding sequences of corresponding amyloid proteins (amyloid precursor protein APP, and alpha-synuclein, respectively). The mutant Aβ peptide, Aβ 40ARCTIC, is more prone to oligomerization than wild-type Aβ 40 [4], whereas PD-associated mutations promote α-synuclein aggregation [3]. These findings suggest that the first model accounts best for the toxicity (the kinetics of aggregate formation would be the key factor). However, mutations leading to highly toxic amyloid would not be found in mammalian since it should lead to lethality early during the developmental process.

In this study, we used a genetic approach to select such harmful amyloid among a randomly mutated library of benign amyloid. This approach permits the exploration of a vast combination of amino acid substitutions without predetermining the events leading to phenotypic changes. The starting material used was a chimeric protein resulting from the fusion between the prion domain of HET-s and the GFP.

None of the mutants isolated caused the death of all yeast cells. The most toxic species (m8) blocks cell growth, but after a lag period, the yeast cells seems to escape and divide as the control cells. This toxicity may be solely due to GFP over expression [36], as opposed to the qualitative effect of its aggregation. This explanation was ruled out, as the level of GFP expression in the m8 toxic mutant was clearly lower than the GFP level in wt yeast cells. The western blot analysis also demonstrates that the toxicity was not due to a particular metabolism of the GFP that would result from a cleavage since the percent of free GFP resulting from proteolysis was roughly the same in m8 and wt extracts. The cells observed with a fluorescence microscope do not differ in fluorescence intensity, but rather in aggregation patterns, as the ring formed by the wt protein is transformed into dotted structures in m8. This structure may reflect a change in intracellular traffic that may be related to a cellular answer involving particular structures such as aggregasomes [37], [38].

Mutations causing growth impairment are found all along the entire coding sequence. Side chain–side chain interaction across neighboring β-strands is a key determinant of amyloid fibril formation and these interactions have been previously predicted by computer analysis software, such as PASTA [39]. Interestingly, the peak encompassing the ß2 strand region [11], [40] dramatically decreases if the m8 mutant is analyzed by PASTA (Figure S3). PASTA is based on an assumption of interchain pairing, but a previous model of Het-s aggregation proposes an intrachain pairing as a packing model. Despite this difference in the proposed models, the amyloid capacity of m8 diverges greatly from that of the wt. This difference may be the basis for the toxic capacity of the m8. However, when we have separated the mutations and generated new alleles that retain only some of them in a wt backbone, we failed to isolate a group of mutations that would be responsible for the toxicity. The two alleles that bear the first four and the last six mutations were harmless. This finding also explains the relative scarcity of toxic mutant since it requires at least two mutations located in each of the two levels of ß-strand turn ß-strand that forms elementary motif of amyloid assembly.

Thus, to understand why m8 is toxic, we analyzed the shape of the cells when this toxicity reaches its maximum. At this stage, m8-GFP is barely detectable on a western-blot and is found equally in the pellet and 100,000 g supernatant. Some of the cells present an unusual shape; the yeast cells (mother and daughter) are still attached to each other. Half of the non-essential genes in yeasts affect cell morphology when they are deleted [41]; and this morphological analysis alone is therefore insufficient for presenting a coherent hypothesis on the molecular events that lead to this phenotype. However, the particular pattern seen in these experiments indicates a loss of polarity, together with a defect in cytokinesis, and identifies various pathways for future investigation.

The cellular aggregation pattern of m8-GFP clearly differs from that of the wild-type HET-s(PrD)-GFP, if observed with a fluorescence microscope. The dotted structures instead of ring aggregates found in stationary phase indicate a greater propensity to be severed or a lower capacity to polymerize. The aggregates formed in both cases are not equivalent at the molecular level, as they do not present similar sensitivity to the mild detergent sarkosyl. This difference indicates either a modification in monomer stacking that is responsible for the fibril formation, or a different interaction with cellular partners that modulate detergent accessibility. Interestingly, these two characteristics were also found for the m4 mutant that is slightly toxic. Moreover non-toxic mutants bearing some m8 mutations exhibit also the same pattern of fluorescence. These level of characterizations are therefore clearly not sufficient to attest the presence of toxic species without any ambiguity.

In the presence of 0.1% sarkosyl, m8 and wt aggregates differ in size. Wild-type aggregates are excluded from the column as a thinner peak and the corresponding species migrate higher on a native gel than m8-GFP aggregates. The presence of PrD-GFP fusions as opposed to GFP alone was confirmed by western-blot in all these fractions. Biochemical differences between m8 and wt PrD-GFP were further confirmed by visually observing yeast total extract after cell lysis. After 24 h of induction, the living cells mostly present a diffuse fluorescence (at this stage, there are no significant differences between wt and m8 cells). After lysis, wt total extract exhibits a thin, branched network of fluorescent proteins, whereas m8 total extract shows dotted fluorescence. Thus, in the stationary phase, these differences may be exacerbated as either a ring or a dot pattern.

Is there any relationship between m8 and wt aggregates? One could imagine that wt-GFP produce toxic species in a lower amount. The difference of toxicity would not be due to the existence of different intermediates but rather to their concentration in each case. In this scenario m8 would be toxic because it produces more of the same toxic species than wt, and thus leads to a strong mortality. If m8 toxicity is due to a higher concentration of a toxic intermediate that would be also present during the wt polymerization, then co-expression of wt and m8 species should change the equilibrium and should attenuate this toxicity. As wt species are more efficiently produced than m8 when the toxicity reaches its maximum, this “titration” should be clearly observed. The introduction of a second plasmid decreases by itself the toxicity, probably by lowering the amount of m8-GFP that is produced (by a simple decrease in the plasmid copy number due to the presence of two plasmids). However, it is quite clear that the residual toxicity is not affected by the presence of the wt-GFP protein. The toxicity of m8 is therefore due to a mechanism apparently independent of wild-type amyloidogenesis and our results favor a model with two aggregation pathways. Moreover our *in vitro* results are consistent with the existence of an alternative pathway since m8 and wt are both amyloids that differ structurally. In addition we did not observe any cross-seeding between these two species [17]. Interestingly, the toxicity was completely abolished in the *Δhsp104* strain. *HSP104* encodes the heat shock protein required for induced thermotolerance [42] and resolubilizing aggregates of denatured proteins [43]. This protein plays a key role in yeast prion propagation [44] and is also involved in the toxicity induced by polyQ aggregation [5]. However, the role of Hsp104 in this latter case is indirect as deleting the *RNQ1* gene specifically suppressed aggregation and toxicity of polyQ. In a *Δhsp104* strain, Rnq1p cannot be transformed in an aggregated prion isoform, making the polyQ non-toxic. In our study, Hsp104 was strictly required for m8 toxicity. Rnq1p was not involved in toxicity, as its deletion did not change the aggregation pattern (observed with a fluorescence microscope). Also, we failed to demonstrate any change in the toxicity induced by the m8 mutant in this strain. The pattern of cellular aggregation of m8 HET-s(PrD)-GFP is different in a *Δhsp104 versus* the wt strain; thus, we favor a model in which Hsp104 modifies the level of aggregation of m8 and, in turn, the level of toxicity.

In conclusion, benign amyloid can be transformed into a toxic species by changing only a few residues. Most toxic effects of deleterious mutant are mediated by small soluble aggregates intermediates in the fibril assembly process. We have shown that the toxic mutant differs from the wild-type in that it is less able to form large and stable aggregates. This link between the polymerization capacity and toxicity support the emerging view that the level of polymerization controls the amyloid toxicity by balancing the concentration of smaller particles. Computational analysis, microscope observation, and biochemical and genetic analysis argue for a model in which the main differences between m8 and wt HET-s(PrD)-GFP are due to an alternative aggregation pathway partially controlled by Hsp104 rather than a difference in the kinetics of polymerization.

Our study opens a new avenue in the field and paves the way of the future by permitting a structure-toxicity approach without any structural pre-requisites. Investigating the differences between m8 and wt PrD aggregates at the molecular level is now necessary to better understand the molecular composition of the toxic species.

## Materials and Methods

### Yeast strains, media and plasmids

Yeast strains used are isogenic to BY4742 (*MATa*, *his3Δ1*, *leu2Δ0*, *ura3Δ0*). Deletion strains are from the Euroscarf yeast deletion strain set [45]: *hsp104Δ* is Y11514 (*MATa*, *his3Δ1*, *leu2Δ0*, *ura3Δ0*, *YLL026w::kanMX4*), *rnq1Δ* is Y13435 (*MATa*, *his3Δ1*, *leu2Δ0*, *ura3Δ0*, *YCL028w::kanMX4*) and *trp1Δ* is Y17202 (*MATa*, *his3Δ1*, *leu2Δ0*, *ura3Δ0*, *YDR007w::kanMX4*).

As specified, yeasts were grown in SD medium (0.67% yeast nitrogen base, 2% dextrose) or SG medium (0.67% yeast nitrogen base, 2% galactose) which were supplemented with 20 mg/L histidine (H), 20 mg/L lysine (K), and 60 mg/L leucine (L).

The wt plasmid used in this study was pYecHetsYGFP2U (wt-GFP). This is a multicopy (*2 µ*) yeast expression plasmid with the *URA3* selectable marker. The fusion of the HET-s(PrD) and yeast optimized *GFP* [13] is expressed under control of a *GAL10* promoter in a pYeHFN2U [46] backbone. This pYeHFN2U empty plasmid was used as control in cell viability studies. The pYecHetsYGFP2T plasmid, expressing wild-type HET-s(PrD)-GFP, was also used in *trp1Δ* strain, this plasmid is similar to the pYecHetsYGFP2U except for the selectable marker which is *TRP1*. *HSP104* bearing plasmids used were derived from pA1/4 (pFL44L+HSP104, *URA3*, *2 µ*) [47] except for the selectable marker which is *LEU2*. The *HSP104* ORF was also isolated and integrated under control of a *GAL10* promoter in a pYeHFN2L [46] backbone.

The m8N and m8C constructions were generated by exchanging N-ter and C-ter part of respectively wt-GFP and m8-GFP plasmids. This was achieved using cloning sites *BamHI*/*PstI*. The m8PZ synthetic gene was from Genscript Corp., USA. The gene was cloned in yeast vector by a gap repair strategy. After a PCR using oligonucleotides pGalWT(+) 5′-CACAAATACACACACTAAATTACCGGATCTATGAAGATCGACGCG-3′ and wt-GFP(−) 5′-CCAGTGAATAATTCTTCACCTTTAGACATATTATCCCGGAACCC-3′, the amplified gene was co-transformed with *BamHI* linearized and Mung-Bean treated (Promega) wt-GFP yeast plasmid.

All yeast transformations were carried out as described previously [48].

### PCR mutagenesis

The HET-s(PrD) sequence of pYecHetsYGFP2U was amplified by PCR using 5′- AAATACACACACTAAATTACCGGATCTATG -3′ and 5′- ACCAGTGAATAATTCTTCACCTTTAGACAT -3′ primers. A Taq DNA polymerase with no proofreading activity (New England BioLabs M0237 Taq DNA polymerase) was used in error-prone reaction conditions (0.1% (v/v) Triton X-100; 50 mM KCL ; 10 mM Tris-HCl [pH 8.3]; 4.76 mM MgSO_4_ ; 0.5 mM MnCl_2_) with the following nucleotide concentrations (0.09 mM dCTP ; 0.06 mM dATP; 0.14 mM dTTP; 0.02 mM dGTP). The corresponding PCR product was cloned by “gap-repair” in a BamHI (New England BioLabs) linearized and Mung-Bean nuclease (New England BioLabs) treated pYecHetsYGFP2U plasmid.

### Isolation of toxic mutants

The library obtained after the gap-repair was platted onto SD HKL medium. After replica plating onto SG HKL medium, colonies presenting a growth defect were isolated and spotted individually. In this study 20 000 independent clones were thus analyzed and 80 were further selected. Only 5 clones were confirmed for a toxic phenotype. Plasmids were then extracted and re-transformed into *S. cerevisiae* after an amplification step in *E. coli*. The interesting clones were finally sequenced.

### Spotting assay

All spotting assays were performed in the same conditions. Tenfold serial dilutions starting with equal number of cells (10^7^ cells) were made in sterile water. Spotting assays derived from a pool of three independent fresh colonies. Five microliters drops were then plated on SD or SG medium complemented with appropriated amino acids.

### Colony Forming Unit assay

Yeasts were grown overnight in SD HKL liquid medium. They were washed in water and then inoculated in 50 mL of liquid SG HKL medium at 0.05 OD (λ = 600 nm) and grown for 65 hours at 30°C with shaking. Aliquots were collected at various times (18 to 65 hours) and diluted before plating onto SD HKL medium to have a proper number of colonies. Colonies were numbered after 2 days of growth at 30°C and this value was converted to colony forming unit (cfu/mL) by applying dilution factor.

### Microscopy

Cells were washed in water and resuspended into a mounting solution (218 mM 1,4-diazabicyclo[2.2.2]octane (DABCO, Sigma); 25% (v/v) PBS 1×; 75% glycerol). DNA was stained by adding 2 ng/mL Hoechst 33342 to the mounting solution. Cells were observed using either a DMLB (Leica) fluorescence microscope coupled with a ColorView II (Olympus) color camera or an Axioskop 2 plus (Zeiss) fluorescence microscope coupled with an AxioCam (Zeiss) black and white camera. The following filters were used: L5 (GFP) and A4 (Hoechst) for the Leica and GFP LP (GFP) for the Zeiss.

### Protein extraction, sedimentation analysis and Western blotting

Total yeast protein extracts were prepared by lysing the equivalent of 20OD (λ = 600 nm) units of yeast cells in exponential growth with glass beads in 200 µL of TNT extraction buffer (25 mM Tris-HCl [pH 7.4], 100 mM NaCl, 0.2% Triton), containing 2 mM phenylmethylsulfonyl fluoride (Genaxis) and some protease inhibitors (Roche Complete™ mini), using a MP Biomedicals FASTPREP®-24 device for 30 seconds. This extract was then centrifuged for 10 min at 1000 g at 4°C and supernatant was recovered.

An alternative alkaline lysis extraction method was also used. Briefly, 5OD (λ = 600 nm) units of yeast cells in exponential growth were permeabilized with 500 µL of 0.185 M NaOH, 0.2% 2-mercaptoethanol. After a 10 min incubation on ice Trichloroacetic acid (TCA) was added to a final concentration of 5%, and the samples were incubated for an additional 10 min on ice. Precipitates were then collected by centrifugation at 14000 g for 5 min. Pellets were dissolved in 30 µL of dissociation buffer (4% sodium dodecyl sulfate, 0.1 M Tris-HCl [pH 6.8], 4 mM EDTA, 20% glycerol, 2% 2-mercaptoethanol, 0.02% bromophenol blue) and 15 µl of 1 M Tris-base. Yeast proteins were incubated for 5 min at 100°C and separated on a 12% SDS-PAGE.

For the western-blot, the crude extract was incubated for 5 min at 100°C in 1× loading buffer and separated on a 12% SDS-PAGE. Proteins were electrically transferred onto nitrocellulose membranes (Optitran BA-S83, Schleicher & Schuell) in the presence of transfer buffer (39 mM Glycine, 48 mM Tris-base, 2% EtOH and 0.037% SDS) and were probed with monoclonal anti-GFP antibodies (Sigma). Peroxidase-conjugated anti-mouse antibodies (Sigma) were used as secondary antibodies. Binding was detected with the SuperSignal reagent (Pierce) and the VersaDoc Imaging system (BioRad). Signals were quantified with Quantity One software (Bio-Rad).

For the sedimentation analysis, the total yeast extract was centrifuged for 30 min at 100,000 g at 4°C.

### Filter trap assay

A cellulose acetate membrane (OE66, Schleicher & Schuell) was equilibrated in TNT extraction buffer for 5 min, followed by the recommended assembly of the 96-well dot-blot system (Minifold I Dot-Blot System, Schleicher & Schuell). Crude extracts were incubated in TNT extraction buffer +0 to 2% sarkosyl (N-Lauroylsarcosine sodium salt, Sigma) for 10 min at room temperature and subsequently filtered through the membrane. GFP fluorescence was detected using a Safe Imager™ blue light (λ = 470 nm) transilluminator and Safe Imager™ amber filter (Invitrogen). The acetate membrane was then probed with anti-GFP antibodies as previously described for a nitrocellulose membrane.

### Size-exclusion chromatography

0.1% sarkosyl was added to crude extracts before a filtration step through a 0.2 µm cellulose acetate filter (Minisart®, Sartorius). The extracts were then processed through the size-exclusion column. The molecular size of the proteins was analysed by chromatography on a FPLC Superose 6 column (Amersham Biosciences) equilibrated with 100 mM Tris-HCl [pH 8]; 150 mM NaCl and 0.1% sarkosyl.

### Native agarose gel

Ten microliters of each size-exclusion resulting fraction (in 0.1% sarkosyl TNT extraction buffer) were incubated in 1× loading buffer (containing only orange G (Sigma); 20% glycerol; TNT extraction buffer) and separated on a 2.5% agarose gel in a Tris-Glycine (1.45 g/L Tris-base (BioRad) ; 7.2 g/L Glycine (BioRad)) running buffer. Proteins were blotted by capillarity onto nitrocellulose membranes (Optitran BA-S83, Schleicher & Schuell) for one night in the presence of transfer buffer (39 mM Glycine, 48 mM Tris-base, 2% EtOH and 0.037% SDS) and then probed with anti-GFP antibodies as previously described for Western blot.

## Supporting Information

Figure S1 Structure of wt HET-s(PrD)-GFP may be conditioned by an asparagine polar zipper. On the RMN predicted structure of HET-s(PrD)-GFP the different beta strands are colorized to show their interactions: beta1, beta3 in red and beta2, beta4 in yellow. Asparagines are visualized by their carbon backbone highlighted in the opposite color.
(0.81 MB TIF)

Figure S2 Intracellular cleavage of GFP is independent of HET-s(PrD)-GFP mutations. Crude extracts were obtained from cells expressing either wt, m4 or m8 proteins either by a glass beads (left) or an alkaline lysis (right) extraction method
(2.85 MB TIF)

Figure S3 Amyloid Propensity Plots for wt and m8 protein as predicted by PASTA algorithm [33]. (A) Plot of amyloid propensity h(k) for the wt protein. Light blue arrows over the k-axis represent the sequence regions involved in β-strands according to ss-NMR experiments. (B) Plot of amyloid propensity h(k) for the m8 protein. Light blue arrows over the k-axis represent the sequence regions involved in β-strands according to ss-NMR experiments on the wild-type protein.
(0.55 MB TIF)

## References

[1] Chiti F, Dobson CM (2006). Protein misfolding, functional amyloid, and human disease.. Annu Rev Biochem.

[2] Lansbury PT, Lashuel HA (2006). A century-old debate on protein aggregation and neurodegeneration enters the clinic.. Nature.

[3] Conway KA, Harper JD, Lansbury PT (1998). Accelerated in vitro fibril formation by a mutant alpha-synuclein linked to early-onset Parkinson disease.. Nat Med.

[4] Nilsberth C, Westlind-Danielsson A, Eckman CB, Condron MM, Axelman K (2001). The ‘Arctic’ APP mutation (E693G) causes Alzheimer's disease by enhanced Abeta protofibril formation.. Nat Neurosci.

[5] Meriin AB, Zhang X, He X, Newnam GP, Chernoff YO, Sherman MY (2002). Huntington toxicity in yeast model depends on polyglutamine aggregation mediated by a prion-like protein Rnq1.. J Cell Biol.

[6] Willingham S, Outeiro TF, DeVit MJ, Lindquist SL, Muchowski PJ (2003). Yeast genes that enhance the toxicity of a mutant huntingtin fragment or alpha-synuclein.. Science.

[7] Outeiro TF, Lindquist S (2003). Yeast cells provide insight into alpha-synuclein biology and pathobiology.. Science.

[8] Cooper AA, Gitler AD, Cashikar A, Haynes CM, Hill KJ (2006). Alpha-synuclein blocks ER-Golgi traffic and Rab1 rescues neuron loss in Parkinson's models.. Science.

[9] Coustou V, Deleu C, Saupe S, Begueret J (1997). The protein product of the het-s heterokaryon incompatibility gene of the fungus Podospora anserina behaves as a prion analog.. Proc Natl Acad Sci U S A.

[10] Balguerie A, Dos Reis S, Ritter C, Chaignepain S, Coulary-Salin B (2003). Domain organization and structure-function relationship of the HET-s prion protein of Podospora anserina.. EMBO J.

[11] Ritter C, Maddelein ML, Siemer AB, Lührs T, Ernst M (2005). Correlation of structural elements and infectivity of the HET-s prion.. Nature.

[12] Wasmer C, Lange A, Van Melckebeke H, Siemer AB, Riek R, Meier BH (2008). Amyloid fibrils of the HET-s(218–289) prion form a beta solenoid with a triangular hydrophobic core.. Science.

[13] Taneja V, Maddelein ML, Talarek N, Saupe SJ, Liebman SW (2007). A non-Q/N-rich prion domain of a foreign prion, [Het-s], can propagate as a prion in yeast.. Mol Cell.

[14] Maddelein ML, Dos Reis S, Duvezin-Caubet S, Coulary-Salin B, Saupe SJ (2002). Amyloid aggregates of the HET-s prion protein are infectious.. Proc Natl Acad Sci U S A.

[15] Perutz MF, Pope BJ, Owen D, Wanker EE, Scherzinger E (2002). Aggregation of proteins with expanded glutamine and alanine repeats of the glutamine-rich and asparagine-rich domains of Sup35 and of the amyloid beta-peptide of amyloid plaques.. Proc Natl Acad Sci U S A.

[16] Perutz MF, Johnson T, Suzuki M, Finch JT (1994). Glutamine repeats as polar zippers: their possible role in inherited neurodegenerative diseases.. Proc Natl Acad Sci U S A.

[17] Berthelot K, Immel F, Géan J, Lecomte S, Oda R (2009). Driving amyloid toxicity in a yeast model: a molecular approach.. FASEB J in press.

[18] Chapman MR, Robinson LS, Pinkner JS, Roth R, Heuser J (2002). Role of Escherichia coli curli operons in directing amyloid fiber formation.. Science.

[19] Jeter C, Matthysse AG (2005). Characterization of the binding of diarrheagenic strains of E. coli to plant surfaces and the role of curli in the interaction of the bacteria with alfalfa sprouts.. Mol Plant Microbe Interact.

[20] Claessen D, Rink R, de Jong W, Siebring J, de Vreugd P (2003). A novel class of secreted hydrophobic proteins is involved in aerial hyphae formation in Streptomyces coelicolor by forming amyloid-like fibrils.. Genes Dev.

[21] Sparrer HE, Santoso A, Szoka CF, Weissman JS (2000). Evidence for the prion hypothesis: induction of the yeast [PSI(+)] factor by in vitro- converted sup35 protein.. Science.

[22] King CY, Diaz-Avalos R (2004). Protein-only transmission of three yeast prion strains.. Nature.

[23] Patel BK, Liebman SW (2007). “Prion-proof” for [PIN+]: infection with in vitro-made amyloid aggregates of Rnq1p-(132–405) induces [PIN+].. J Mol Biol.

[24] Brachmann A, Baxa U, Wickner RB (2005). Prion generation in vitro: amyloid of Ure2p is infectious.. EMBO J.

[25] Wickner RB (1994). [URE3] as an altered URE2 protein: evidence for a prion analog in Saccharomyces cerevisiae.. Science.

[26] Solomon A, Richey T, Murphy CL, Weiss DT, Wall JS (2007). Amyloidogenic potential of foie gras.. Proc Natl Acad Sci U S A.

[27] Zhang B, Une Y, Fu X, Yan J, Ge F (2008). Fecal transmission of AA amyloidosis in the cheetah contributes to high incidence of disease.. Proc Natl Acad Sci U S A.

[28] Nakayashiki T, Kurtzman CP, Edskes HK, Wickner RB (2005). Yeast prions [URE3] and [PSI+] are diseases.. Proc Natl Acad Sci U S A.

[29] Ganusova EE, Ozolins LN, Bhagat S, Newnam GP, Wegrzyn RD (2006). Modulation of prion formation, aggregation, and toxicity by the actin cytoskeleton in yeast.. Mol Cell Biol.

[30] Tyedmers J, Madariaga ML, Lindquist S (2008). Prion switching in response to environmental stress.. PLoS Biol.

[31] Kayed R, Head E, Thompson JL, McIntire TM, Milton SC (2003). Common structure of soluble amyloid oligomers implies common mechanism of pathogenesis.. Science.

[32] Sanbe A, Osinska H, Saffitz JE, Glabe CG, Kayed R (2004). Desmin-related cardiomyopathy in transgenic mice: a cardiac amyloidosis.. Proc Natl Acad Sci U S A.

[33] Shorter J, Lindquist S (2004). Hsp104 catalyzes formation and elimination of self-replicating Sup35 prion conformers.. Science.

[34] Baglioni S, Casamenti F, Bucciantini M, Luheshi LM, Taddei N (2006). Prefibrillar amyloid aggregates could be generic toxins in higher organisms.. J Neurosci.

[35] Takahashi T, Kikuchi S, Katada S, Nagai Y, Nishizawa M, Onodera O (2008). Soluble polyglutamine oligomers formed prior to inclusion body formation are cytotoxic.. Hum Mol Genet.

[36] Liu HS, Jan MS, Chou CK, Chen PH, Ke NJ (1999). Is green fluorescent protein toxic to the living cells?. Biochem Biophys Res Commun.

[37] Kaganovich D, Kopito R, Frydman J (2008). Misfolded proteins partition between two distinct quality control compartments.. Nature.

[38] Wang Y, Meriin AB, Zaarur N, Romanova NV, Chernoff YO (2008). Abnormal proteins can form aggresome in yeast: aggresome-targeting signals and components of the machinery.. FASEB J.

[39] Trovato A, Seno F, Tosatto SC (2007). The PASTA server for protein aggregation prediction.. Protein Eng Des Sel.

[40] Trovato A, Chiti F, Maritan A, Seno F (2006). Insight into the structure of amyloid fibrils from the analysis of globular proteins.. PLoS Comput Biol.

[41] Ohya Y, Sese J, Yukawa M, Sano F, Nakatani Y (2005). High-dimensional and large-scale phenotyping of yeast mutants.. Proc Natl Acad Sci U S A.

[42] Sanchez Y, Lindquist SL (1990). HSP104 required for induced thermotolerance.. Science.

[43] Parsell DA, Kowal AS, Singer MA, Lindquist S (1994). Protein disaggregation mediated by heat-shock protein Hsp104.. Nature.

[44] Chernoff YO, Lindquist SL, Ono B, Inge VS, Liebman SW (1995). Role of the chaperone protein Hsp104 in propagation of the yeast prion- like factor [psi+].. Science.

[45] Winzeler EA, Shoemaker DD, Astromoff A, Liang H, Anderson K (1999). Functional characterization of the S. cerevisiae genome by gene deletion and parallel analysis.. Science.

[46] Cullin C, Minvielle-Sebastia L (1994). Multipurpose vectors designed for the fast generation of N- or C-terminal epitope-tagged proteins.. Yeast.

[47] Chacinska A, Boguta M, Krzewska J, Rospert S (2000). Prion-dependent switching between respiratory competence and deficiency in the yeast nam9-1 mutant.. Mol Cell Biol.

[48] Gietz RD, Schiestl RH, Willems AR, Woods RA (1995). Studies on the transformation of intact yeast cells by the LiAc/SS- DNA/PEG procedure.. Yeast.

