# Supplementary figures and images for: Screening for Toxic Amyloid in Yeast Exemplifies the Role of Alternative Pathway Responsible for Cytotoxicity
Source: PLoS One. 2009 Mar 5;4(3):e4539. doi: 10.1371/journal.pone.0004539 (PMC2650408; doi:10.1371/journal.pone.0004539)

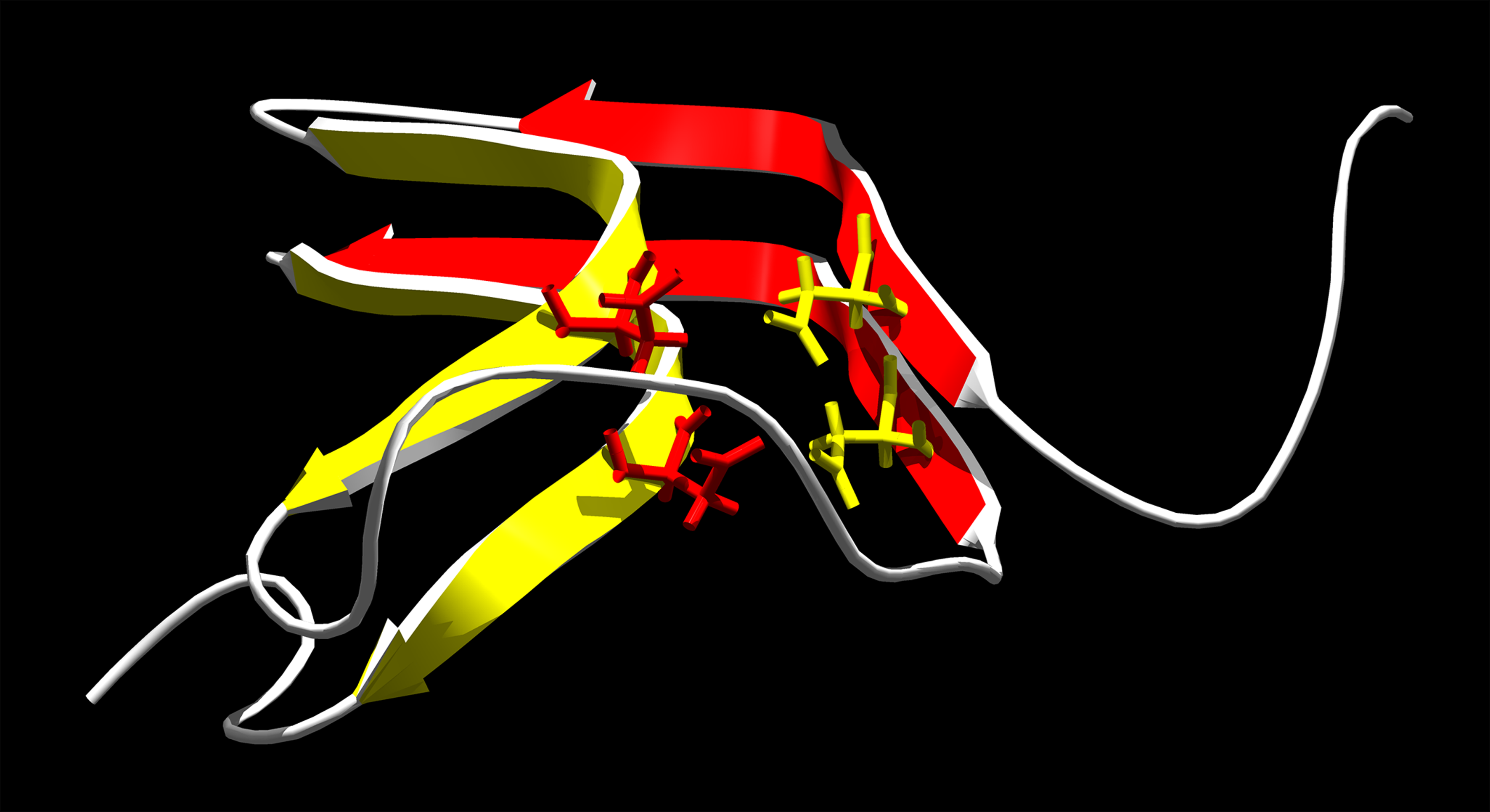

Supplement: Figure S1 — Structure of wt HET-s(PrD)-GFP may be conditioned by an asparagine polar zipper. On the RMN predicted structure of HET-s(PrD)-GFP the different beta strands are colorized to show their interactions: beta1, beta3 in red and beta2, beta4 in yellow. Asparagines are visualized by their carbon backbone highlighted in the opposite color. (0.81 MB TIF) [file pone.0004539.s001.tif]

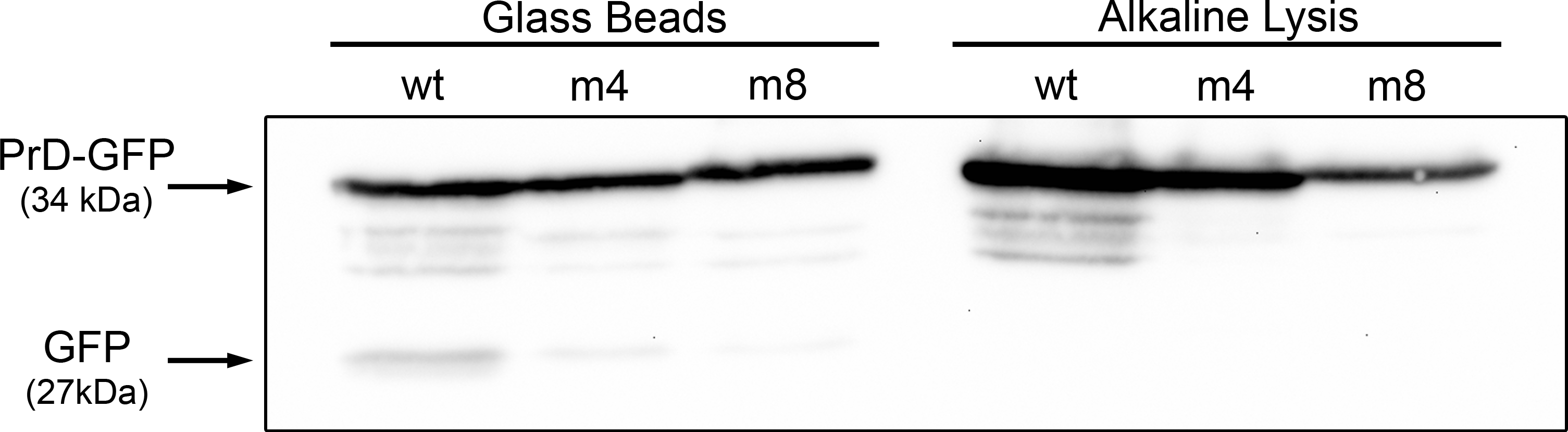

Supplement: Figure S2 — Intracellular cleavage of GFP is independent of HET-s(PrD)-GFP mutations. Crude extracts were obtained from cells expressing either wt, m4 or m8 proteins either by a glass beads (left) or an alkaline lysis (right) extraction method (2.85 MB TIF) [file pone.0004539.s002.tif]

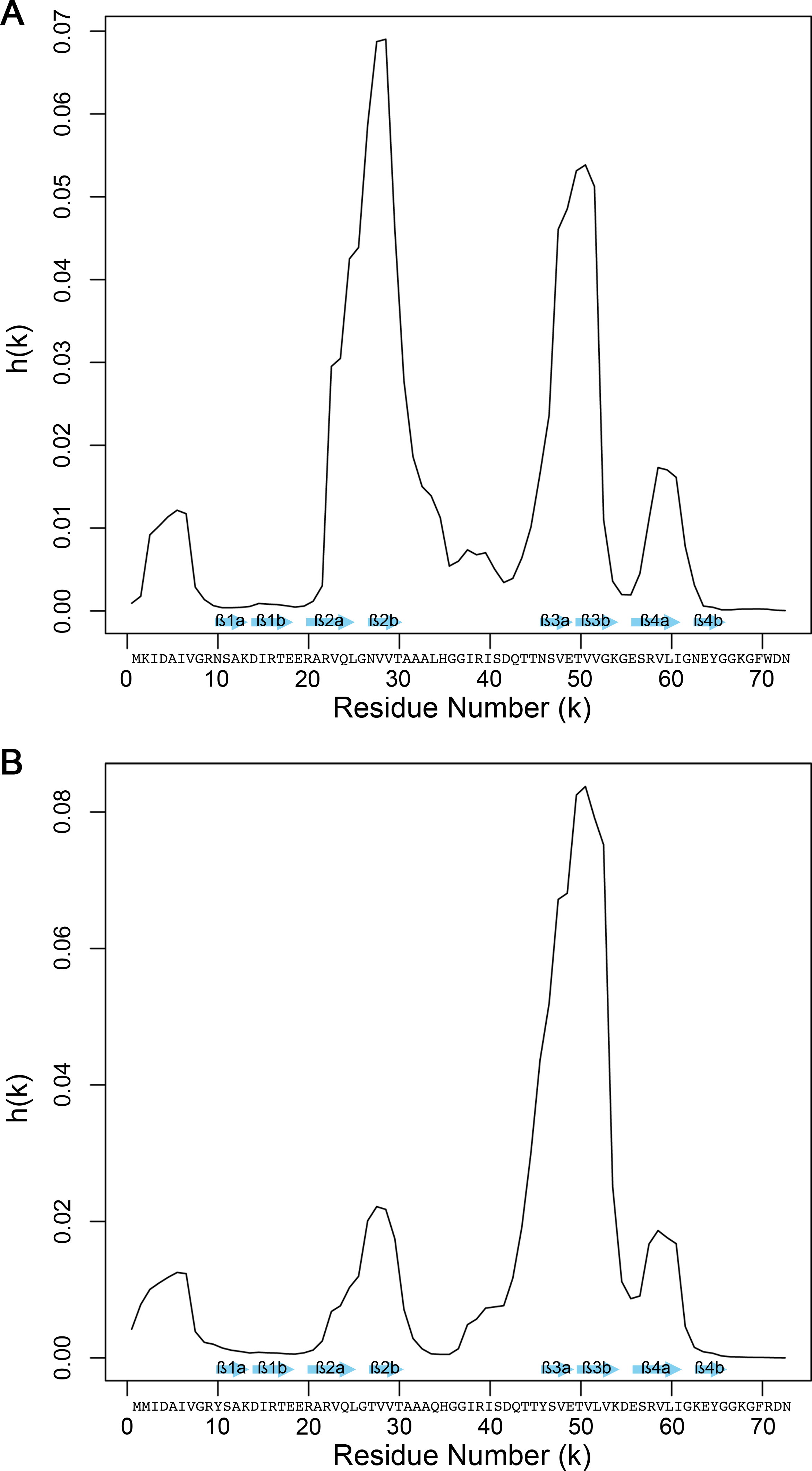

Supplement: Figure S3 — Amyloid Propensity Plots for wt and m8 protein as predicted by PASTA algorithm [33]. (A) Plot of amyloid propensity h(k) for the wt protein. Light blue arrows over the k-axis represent the sequence regions involved in β-strands according to ss-NMR experiments. (B) Plot of amyloid propensity h(k) for the m8 protein. Light blue arrows over the k-axis represent the sequence regions involved in β-strands according to ss-NMR experiments on the wild-type protein. (0.55 MB TIF) [file pone.0004539.s003.tif]
